# Intracranial neuroendocrine tumour simulating meningioma for several years: an overview of diagnosis and treatment

**DOI:** 10.1259/bjrcr.20210222

**Published:** 2022-01-27

**Authors:** Mohamed Badawy, Geoffrey Johnson, Manoj Jain, Ayse Tuba Kendi, Derek R. Johnson, Alyx Porter, Ming Yang, Mohamad Bassam Sonbol

**Affiliations:** 1 Division of Radiology and Nuclear Medicine, Mayo Clinic, Rochester, MN; 2 Department of Immunology, Mayo Clinic, Rochester, MN; 3 Division of Radiology and Nuclear Medicine, Mayo Clinic, Jacksonville, Florida, USA; 4 Division of Neurology, Mayo Clinic, Rochester, MN; 5 Division of Neurology, Mayo Clinic, Phoenix, Arizona; 6 Division of Diagnostic Radiology, Mayo Clinic, Phoenix, Arizona; 7 Division of Hematology- Medical Oncology, Mayo Clinic, Phoenix, Arizona

## Abstract

Metastatic neuroendocrine tumour (NET) to brain has been reported in 1.5–5% of patients with NETs. Differentiation between intracranial NET metastasis and meningiomas can cause a diagnostic dilemma. We present a symptomatic case of a 66-year-old male with a history of left-sided skull base mass. The diagnosis of a meningioma was made based on the MRI findings and clinical presentation. The patient received radiation and the mass remained stable on serial MRI images at follow-up visits. Five years after his initial presentation, the patient’s mass showed further growth. He also complained of worsening of his recent diagnosis of irritable bowel syndrome and fluctuations in his blood pressure. Surgical resection was performed, and histopathological features were consistent with moderately differentiated neuroendocrine tumour. Further evaluation with 68 Gallium-DOTATATE positron emission-computed tomography (Ga-68 PET/CT) showed metastatic disease involving the bones, lymph nodes, and liver without convincing evidence of the location of primary malignancy within the bowel loops or the pancreas. The patient was started on combination of capecitabine and temozolomide with partial response and significant improvement of his symptoms. This case highlights the clinical and radiological behaviour of intracranial NET that can mimic the diagnosis of meningioma.

## Introduction

The incidence of neuroendocrine tumors (NETs) has increased in the United States over the last two decades.^1^ NETs arise from enterochromaffin cells that are widely distributed all over the body.^2^ Gastrointestinal and respiratory tracts are the most common primary sites of NETS.^3^ Most metastases occur in the liver, bones, and lungs. NETs can rarely metastasize to the brain with an incidence less than 5% of patients.^4^ Since meningioma is the most common type of primary intracranial tumors,^5^ differentiating between a dural metastasis and a meningioma is crucial for patient management.

Herein, we present a case of intracranial NET metastasis that mimics a meningioma in a patient without known history of NET at the time of diagnosis.

## Case presentation

**Figure 1.**
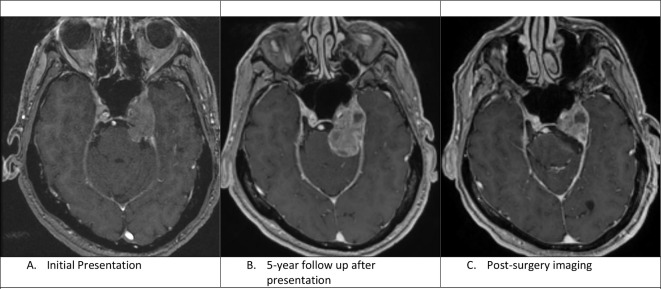
A: Axial C+ T1-weighted image shows an enhancing mass centred in the left cavernous sinus with mild displacement of the medial left temporal lobe. B: Axial C+ T1 weighted image shows interval enlargement of the prepontine component of the mass. There are also some new cystic changes within the mass. C. Axial C+ T1-weighted image shows residual enhancing mass lesion after resection of the cisternal component of the mass.

A 66-year-old, right-handed male presented to the neurology clinic with intermittent paresthesia over the left cheek and chin region. In addition, he experienced an intermittent double vision that was worsening in the 3 months prior. Magnetic resonance imaging (MRI) of the brain showed an enhancing mass centered in the left cavernous sinus, with extension to the brainstem homolaterally, causing mild displacement of the medial left temporal lobe without evidence of brain oedema. The mass was infiltrating the left Meckel’s cave. The clinical and radiological findings were consistent with a meningioma (Figure 1).

After multidisciplinary discussion, stereotactic radiotherapy was recommended over surgical resection given the location of the tumour with proximity to carotid artery and cranial nerves, making complete surgical resection a challenge. Therefore, the patient received a total of 5040 cGy of radiation over 28 fractions.

During the 6-month follow-up, the patient had significant improvement of his diplopia and facial dysesthesias. Follow-up MRIs over the next 3 years showed stable appearance of the mass, and the patient showed complete clinical neurological recovery. However, almost 5.5 years after radiation therapy, a repeat MRI scan revealed significant enlargement of the prepontine and the cavernous sinus lesion with no associated neurological symptoms (Figure 1). On review of systems, the patient reported major fluctuations of blood pressure over the few months prior, accompanied by a new diagnosis of irritable bowel syndrome that manifested with persistent diarrhoea.

**Figure 2.**
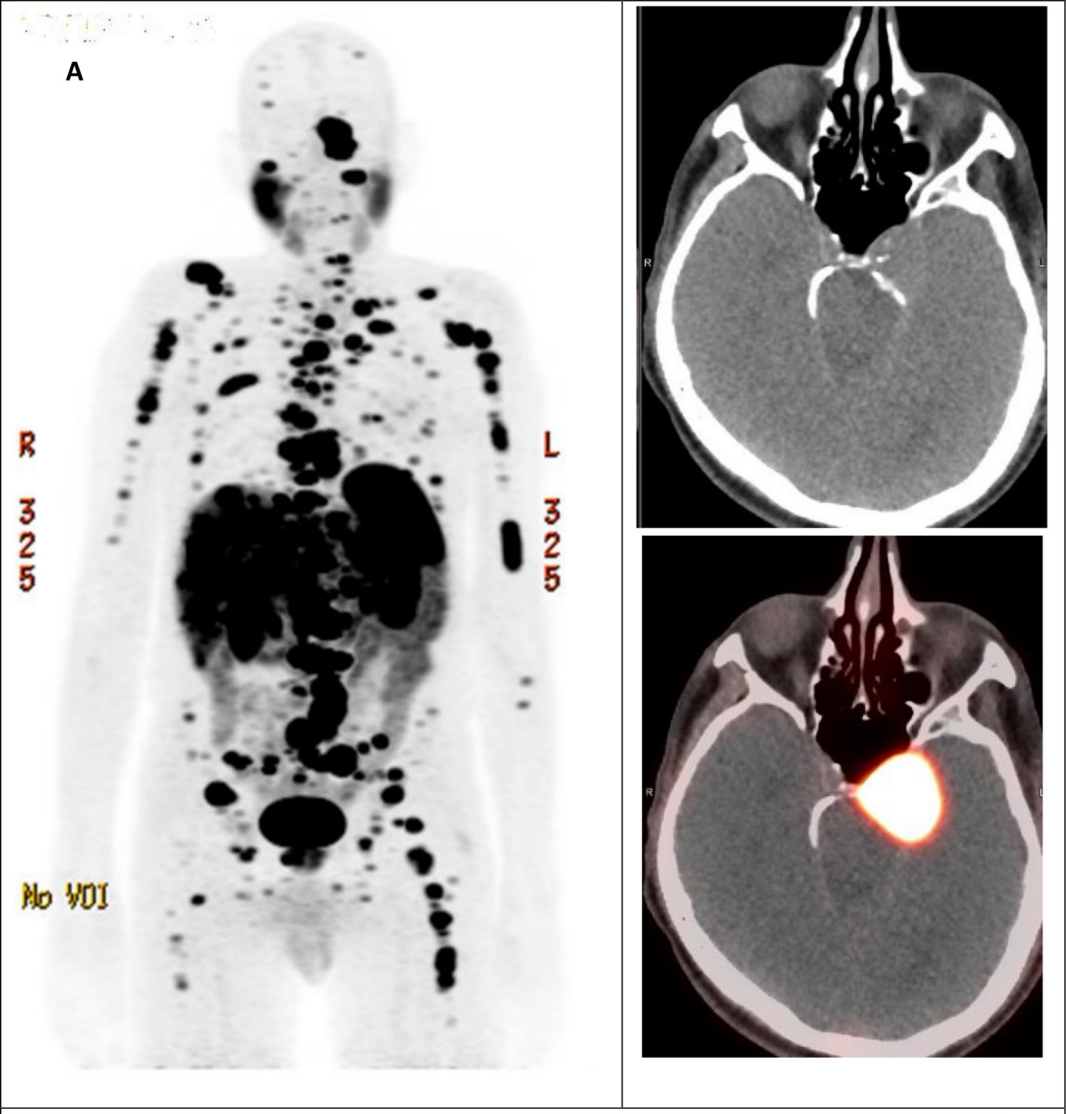
A. Gallium68-DOTATATE PET/CT MIP image shows numerous tracer avid lesions involving the skeleton, lymph nodes and liver, compatible with somatostatin receptor positive lesions. (B, C) The residual left skull base lesion demonstrates intense tracer uptake with SUVmax 29.0. MIP: Maximal Intensity Projection. SUV: Standard Uptake Value.

After multidisciplinary team discussion, the decision of partial surgical resection was recommended due to brain stem compression. The patient underwent resection of his prepontine mass that had caused brainstem compression with residual mass located at the cavernous sinus. He showed a reasonable post-operative recovery, although he had some difficulties such as persistent ataxia and syncope-like episodes. Surprisingly, the pathology came back as well-differentiated neuroendocrine tumour, with Ki 67 index of 10% and diffusely strong positive immunostaining for synaptophysin, chromogranin, and CDX2. The patient was referred to the neuroendocrine oncology clinic. 68 Gallium-DOTATATE positron emission-computed tomography (Ga-68 PET/CT) scan evaluation revealed metastatic lesions affecting liver, bones, and lymph nodes without evidence of primary tumour (Figure 2). The patient was started on capecitabine and temozolomide (CAPTEM) in addition to long-acting octreotide.

The patient’s mass continued to show response on CAPTEM with partial response in the liver lesions and stable MRI appearance and no evidence of new metastatic lesions. On the patient’s last visit (20 months post-operative and seven and half years post his first radiation therapy), he denied any new seizures, headache, or cognitive dysfunction. He showed few symptoms of peripheral neuropathy without any neuropathic pain or subsequent falls. The patient is completely independent in all his daily living activities.

## Discussion

In this report, we present a case of metastatic NET to the dura with radiological features suggestive of meningioma. The majority of NETs arise in the gastrointestinal tract or in the bronchopulmonary system. Most metastases occur in the liver, bones and, lungs, while intracranial metastases are extremely rare.^6^ Only 1.3–1.4% of all brain metastases have neuroendocrine tumour origin.^7–9^ Bronchopulmonary NET is the most frequent subtype to metastasize to the brain.

Dural metastases in general are not uncommon. Moreover, radiological features of many tumours including dural metastases on CT/MRI imaging are often similar to that of meningioma resulting in a diagnostic dilemma.^10^ Stereotactic biopsy should be recommended in cases that the tumours location allows any neurosurgical operation. Otherwise, repeat MRI imaging to show stability or slow growth over 6 months to a year or systemic imaging with CT or MRI to rule out metastatic disease may be helpful if the diagnosis of meningioma is in doubt. However, low-grade NET metastases may grow at a similar slow rate as is seen with meningiomas, thus in this rare case repeat MRI may have falsely generated confidence in the diagnosis of meningioma. Fludeoxyglucose (FDG) uptake on PET imaging is not useful, due to high physiological baseline uptake of the brain.^11^ However, FDG PET is a very useful modality for evaluation of metastatic disease in general.

The national comprehensive cancer network guidelines recommend considering Somatostatin receptor imaging (SRI), such as octreotide planar and SPECT scans or 68 Gallium-DOTATATE PET scans if diagnostic doubt regarding the diagnosis of meningioma exists based on MRI features.^12^ This is because meningiomas routinely express somatostatin receptors and are universally positive on SRI.^13^ For this reason, SRI is known to be useful in distinguishing residual meningioma tumour from postoperative scarring in the post-operative setting.^14,15^ However, SRI can show moderate-to-high activity in a variety of tumour types that are somatostatin-receptor positive, such as NETs..^16^ Therefore, while SRIs might not distinguish NET from meningioma, the presence of extracranial somatostatin receptor positive lesions would point to a diagnosis other than meningioma.

One of the radiotracers that may be, surprisingly, useful for differentiating meningiomas from other tumours is Pittsburgh compound B (11C-PiB) which is used in PET imaging in dementia. We have previously reported a 100% specificity for meningioma with (11C-PiB), although further research is required before this technique becomes part of clinical practice.^17^

Functional NETs secrete hormones that could result in variety of symptoms such as watery diarrhoea, labile blood pressure, and flushing, also known as carcinoid syndrome. The presence of such symptoms can help in the diagnosis of an indeterminate intracranial mass. However, there is often a long interval between the start of symptoms and diagnosis.^18^ Deshaies et al also reported a case of meningioma-like mass that turned out to be NET.^19^ In both our case and their case, carcinoid symptoms were present. This should maintain a high index of suspicion for NET in any patient with carcinoid like symptoms and an intracranial mass. Clinical suspicion should be raised in any intracranial mass including a dural-based lesion or a benign-looking mass that has undergone surveillance for several years. Imaging of NETs is often challenging and usually requires a combination of both anatomic and functional techniques.^20^ Metastasis of carcinoid tumour to intracranial meningioma is also a possibility and has been reported in the literature^21^

In our case, the positive CDX2 seen on pathology that is usually seen in gastrointestinal and pancreatobiliary tract tumours, raised the suspicion of the diagnosis of an intracranial metastasis of unknown origin. This concern was further raised when extensive systemic metastases were seen on imaging. Grade 1/2 NET patients with brain metastasis have an estimated median survival of 15 months from the time of brain metastases diagnosis.^22^ Our patient’s last follow-up was around 8 years following his initial presentation with dural metastasis. Despite the relative short median survival time of patients with intracranial NET metastases, longer survival over five years has been reported in a few cases.^22–24^ This prolonged survival may be partially explained by the well-differentiated subtype of the tumours of those who lived longer^22^

There is still no consensus regarding the standard treatment of intracranial NETs. Treatment options include surgical resection, somatostatin analogs including 177 Lutetium DOTATATE, whole-brain radiation or intensity-modulated radiation therapy. A prior study suggested comprehensive treatment including surgical resection and chemo-radiotherapy is expected to offer a longer tumour-free survival than surgical treatment alone.^25,26^ Our patient showed stabilised intracranial tumour and shrinkage of his liver metastases on his temozolomide and capecitabine treatment. Temozolomide can cross-blood brain barrier and it has shown a valuable effect in treating NETs either alone or in combination with capecitabine.^6,27^ This may highlight the clinical benefit of temozolomide in patients with brain metastases from NETs.^22^ Several factors should be taken into consideration in tailoring the treatment lines including patient age, neurological state, histological differentiation, tumour extension location of the tumour, and its relation to the surrounding structures (*e.g.,* cranial nerves and cavernous sinus). Further research may be needed in prioritising the different treatment lines.

## Conclusion

Intracranial NETs metastases are extremely rare. Carcinoid like symptoms and advanced imaging techniques could help in differentiating neuroendocrine metastases from meningioma. Multidisciplinary care should tailor a comprehensive treatment for patients with intracranial NETs for the best possible outcomes.

## Learning points

- Dural neuroendocrine tumour (NET) metastasis is extremely rare, and its differentiation from meningioma can cause a diagnostic dilemma.
- Clinical suspicion should include intracranial benign-looking mass; even that had undergone surveillance for several years, in patients with watery diarrhoea, flushing, or labile blood pressure symptoms.
- While Somatostatin receptor imaging might not distinguish NET from meningioma, the presence of extracranial somatostatin receptor positive lesions would point to a diagnosis other than meningioma. Pittsburgh compound B may play a promising role in the future.
- Multidisciplinary care should tailor a comprehensive treatment for patients with intracranial NETs for the best possible outcomes.
